# Practice of lingual orthodontics and practitioners’ opinion and experience with lingual braces in the United States

**DOI:** 10.4317/jced.58328

**Published:** 2021-08-01

**Authors:** Heidi H. Huh, Kishore Chaudhry, Richard Stevens, Karthikeyan Subramani

**Affiliations:** 1 Roseman University of Health Sciences, College of Dental Medicine, Henderson, NV, USA

## Abstract

**Background:**

A survey was done on practicing Orthodontists in the United States on their experience with lingual orthodontics. The objectives of this survey study were to assess 1) the satisfaction level with cases treated with lingual orthodontics, 2) factors that influence clinicians’ decision to utilize or not utilize lingual braces in their current practices, and 3) intention of using lingual braces in their future practices, if not used currently, in the U.S.

**Material and Methods:**

A survey questionnaire was electronically distributed to 2,200 active U.S. members of the American Association of Orthodontists (AAO).

**Results:**

85 orthodontists completed the survey. About 25% of respondents practiced lingual orthodontics. Direct mentorship was the most common approach used by orthodontists to learn lingual technique. The most used lingual system among the clinicians that use lingual braces was INBRACE® (34.6%). All respondents were either satisfied or very satisfied with their treatment outcome of cases treated with lingual braces. Improved esthetics and practice differentiation were perceived to be the biggest advantages of practicing lingual orthodontics. Biggest challenges with lingual orthodontics were found to be patient discomfort, cost, longer chair time and technical difficulties. Most common reason for not using lingual braces was technical difficulty, followed by availability of alternative appliances, lack of demand and patient discomfort. Approximately, 70% of those that did not use lingual orthodontics in their current practices responded that they were very likely to incorporate lingual orthodontics in their future practices.

**Conclusions:**

Overall outcome satisfaction level with cases treated with lingual braces was high among the orthodontists that practiced lingual orthodontics. There seemed to be a strong interest in incorporating lingual orthodontics in future practices by clinicians that did not use lingual braces in their current practices. Some of the factors that influenced clinicians’ decision to practice lingual orthodontics were improved esthetics, practice differentiation and increased case acceptance. Technical difficulties, availability of alternative appliances, lack of demand and patient discomfort were some of the factors that were identified to have influenced practitioners’ decision to not offer lingual orthodontics in their current practices.

** Key words:**Orthodontic brackets, Lingual braces, Lingual orthodontics.

## Introduction

Since the introduction of plastic brackets in the early 1970s (1), there has been continuous efforts to improve the esthetics of orthodontic appliances. Especially with the increase in number of adult patients seeking orthodontic treatment, availability of esthetic orthodontic appliance options seems to be a crucial marketing strategy of orthodontic practices, since many adult patients resist wearing traditional metal brackets (2-4). Many esthetic appliances like lingual braces, ceramic brackets, clear aligners and coated esthetic archwires are currently available with their own advantages and disadvantages. However, lingual braces currently provide the ultimate esthetic advantages of being inconspicuous and capable of performing orthodontic tooth movements similar to traditional labial brackets (5,6).

Lingual braces were first introduced by Dr. Kinya Fujita in Japan and Dr. Craven Kurtz in the United States in 1970s (5,7,8). Initial popularity of this new appliance was short lived due to various reasons, including technical and postural challenges of operators, substandard outcomes due to poor understanding of mechanics, inaccuracy and complexity of bonding, patient discomfort and introduction of ceramic brackets as a more predictable alternative esthetic appliance. Over the years, significant efforts have been made to develop lingual biomechanics, bracket designs, wire properties, and manufacturing processes including CAD-CAM (Computer-Aided Design-Computer-Aided Manufacture) based customized lingual appliances and robotic wire bending to address the aforementioned problems (5-9).

Despite increasing demand for esthetic orthodontic appliances and advancement in lingual techniques, recent orthodontic practice studies report that 15% of the United States orthodontic practices use lingual braces, which is a significant decline from 35% in 2015 (10,11). Yet, factors that influence clinicians to embrace or resist this treatment modality in the U.S have not been investigated.

Therefore the purpose of this survey study was to identify 1) the satisfaction level with cases treated with lingual orthodontics, 2) the factors that influence clinician’s decision to use or not use lingual braces in their current practice, and 3) if not used, the intention of using lingual braces in their future practices.

## Material and Methods

A survey questionnaire was developed on a secure online survey platform, Qualtrics (www.qualtrics.com). A pilot survey was conducted among the orthodontic faculty of Roseman University of Health Sciences. The study was approved by the Roseman University of Health Sciences Institutional Review Board. The questionnaire was reviewed and approved by American Association of Orthodontists (AAO) Partners in Research. The link to the survey and a cover letter explaining the objectives were distributed to 2,220 active U.S. AAO members electronically through email. A reminder email was sent after 2 weeks and the survey data was collected over 2 months.

The collected data was analyzed with IBM® SPSS® version 25. Active U.S. orthodontists who are a member of AAO, including orthodontic faculty, were included in the analysis. Retired orthodontists and orthodontic residents were excluded from the study. Descriptive statistics were used to analyze the frequency of response regarding use of lingual braces in terms of 1) practitioner’s information 2) method of introduction, 3) percentage of patients treated, 4) type of cases treated, 5) type of lingual system used, 6) outcome satisfaction level, and 7) reported advantages and challenges with lingual braces. Reasons for not using lingual braces and percentage of respondents who are willing to reconsider was also calculated. Association between participant demographics and the use of lingual braces was assessed using chi-square test.

## Results

A total of 85 responses were recorded. No response was eliminated from data analysis. Fifty-four male and 31 female orthodontists participated in this survey. The highest number of responses came from the age group of 56-65 years (42.4%) and those who have been in practice for 26-35 years (41.2%). Majority of the respondents were practice owners or partners (81.1%) practicing primarily in suburban area (62.4%), which was consistent across the groups that used or did not use lingual braces.

**Figure 1:**
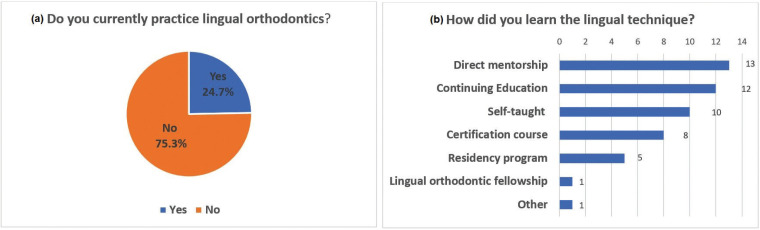
a. Percentage orthodontists that currently use and not use lingual braces in their practices. About 25% of the orthodontists that participated in this survey reported that they currently practiced lingual orthodontics, while the other 75% did not. b. Method of introduction to lingual techniques*. Direct mentorship was the most common approach used by the orthodontists to learn the lingual technique.

About 25% of the orthodontists that participated in this survey reported that they currently practiced lingual orthodontics, while the other 75% did not (Fig. 1a). Among 21 orthodontists who practiced lingual orthodontics, there was 1 clinician who practiced for less than a year, 7 clinicians practicing for 1-5 years, 4 clinicians practicing for 6-10 years and 9 clinicians who have been practicing for more than 10 years. Clinicians who practiced lingual orthodontics the most were found in age groups of 36-45 years and 56-65 years. These 21 clinicians were asked with follow up questions in the same survey questionnaire to select all means used to learn the lingual technique. Direct mentorship was the most common approach by the orthodontists to learn the lingual technique, with 13 responses, followed by 12 responses for continuing education course, 10 responses for self-teaching and 8 responses for certification course, 5 clinicians were taught lingual orthodontics during residency program, 1 was trained by their lingual orthodontic company and only 1 responded that they completed a lingual orthodontics fellowship. Other 1 response included training by lingual orthodontic company (Fig. 1b).

**Figure 2:**
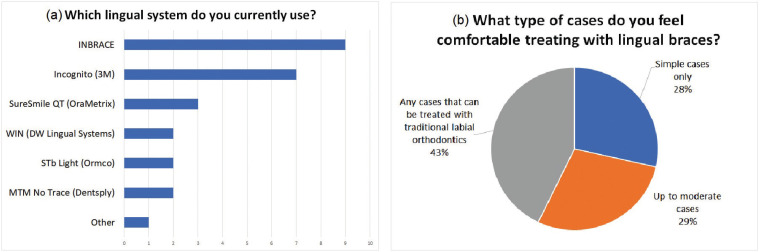
a. Lingual systems that practitioners currently use in their practices*. Most used lingual system among the clinicians that use lingual braces was INBRACE® (34.6%) *Respondents were instructed to select all applicable responses. b. Types of cases treated with lingual braces. Simple cases (e.g. Class I, non-extraction, minor crowding up to 4mm, etc.). Moderate cases (e.g. Extractions, mild to moderate skeletal discrepancy, etc.).

Among the participants that used lingual braces, the majority (76.2%) selected that they treated less than 10% of their total cases with lingual braces, and the age group that was treated with lingual braces the most was found to be the patients between ages 21 to 40 years. Participants in this group were then asked to select all lingual systems that they used in their current practices. The most frequently used lingual system was INBRACE® (34.6%), followed by 3M™ Incognito™ (26.9%) (Fig. 2a). Largest number of orthodontists (43%) in the group that used lingual braces felt comfortable in treating any type of cases that can be treated with traditional labial orthodontics, while some were comfortable treating up to simple cases (28%) and moderate cases only (29%) (Fig. 2b).

**Figure 3:**
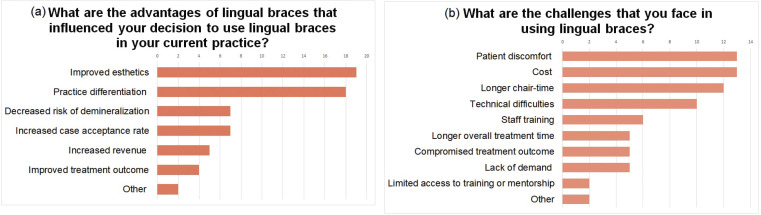
a. Advantages of lingual braces that influenced the decision of practitioners to use lingual braces in their current practices, in decreasing order of frequency of response*. (*Respondents were instructed to select all applicable responses). b. Challenges with lingual orthodontics in decreasing order of frequency of response*.

All respondents who practiced lingual orthodontics were either satisfied or very satisfied with the outcome of cases that were treated with lingual braces. When these respondents were instructed to select all advantages and challenges with practicing lingual orthodontics, improved esthetics and practice differentiation were perceived as advantages of practicing lingual orthodontics by the largest number of participants that use lingual braces (Fig. 3a). On the other hand, patient discomfort, cost, longer chair time and technical difficulties were the four most recognized challenges in lingual orthodontics in this group (Fig. 3b).

**Figure 4:**
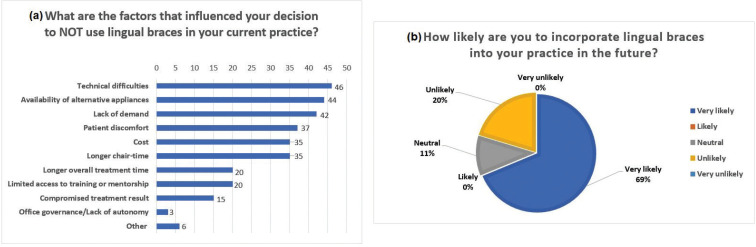
a. Factors that influenced the decision of practitioners to not use lingual braces in their current practice in decreasing order of frequency of response. b. Intention of incorporating lingual brace in the future. Approximately 70% of those that did not used practice lingual orthodontics in their current practices responded that they were very likely to incorporate lingual orthodontics in their future practices.

When participants that did not practice lingual orthodontics were instructed to select all factors that influenced their decision to not use lingual braces in their current practices, technical difficulty was found to be the most common reason, followed by availability of alternative appliances, lack of demand and patient discomfort (Fig. 4a). When asked the likelihood of incorporating lingual braces into their practices in the future, 69% of this group responded very likely, 20% responded unlikely and 11% were neutral (Fig. 4b).

Age, years in practice, and location of practice of respondents had no statistically significant association with the practice of lingual orthodontics.

## Discussion

Our study found that the number of orthodontists who practiced lingual orthodontics in their practice to be higher than the number presented in an orthodontic practice study in 2017 (11). This discrepancy may be due to the small number of respondents, which is a limitation of our study. Comparing the number internationally, prevalence of use of lingual braces recorded in AAO database is comparable to the number published in British Dental Journal in 2018, which reported that 35% of British Orthodontic Society (BOS) members offered lingual braces in their practices (12). However, numbers are lower in the U.S. when compared to the number found in a survey study from India, which showed that 70% of orthodontists in India practiced lingual orthodontics in 2019 (13). This may suggest that the popularity of lingual system in the U.S. has remained low compared to other parts of the world.

Of the many available lingual systems, INBRACE® was most widely used by the participants of this study in their current practices, followed by 3M™ Incognito™, as shown in Figure 2a. Participants were given an option to select multiple response, if more than one lingual system was used. In this study, fully customized lingual systems were a more popular modality compared to prefabricated systems among the orthodontists that used lingual braces. Regardless of the lingual system preferred, all respondents who practiced lingual orthodontics were either satisfied or very satisfied with the outcome of the cases treated with lingual braces.

With the early lingual systems, practitioners were faced with three main challenges – bonding, finishing and patient discomfort. First, indirect visualization and anatomical variation of lingual tooth surfaces complicated precise bonding. A high rate of bonding failure and inaccurate rebonding led to clinical inefficiency as well as poor clinical outcomes. Second, optimal finishing was time-consuming and difficult to achieve due to inaccurate bonding, torque play, as well as short inter-bracket distance. Lastly, prefabricated brackets required the use of composite filler between the bracket base and the tooth surface which increased patient discomfort. Common problems such as speech disturbances, tongue irritation, and masticatory difficulties resulted from the high bracket profile (7,9).

Development of fully customized lingual systems using CAD-CAM technology has overcome major drawbacks associated with traditional lingual orthodontics. Bracket bases can be customized to precisely adapt to the lingual surfaces of each tooth, resulting in more accurate bonding, reduced bond failure, with an optimal finish being achieved more efficiently. Accurate bracket slot production and individualized archwires have also significantly reduced chairside archwire adjustments and contributed to improved efficiency and enhanced clinical outcomes. Fully customized brackets are designed to have a lower profile which minimizes the patient discomfort associated with lingual braces (5,7,9).

Several studies have noted that the advancement in lingual systems have reduced limitations and allowed clinicians to successfully treat any case they would treat with traditional labial systems to a satisfactory level, including those combined with Herbst appliance and orthognathic correction of skeletal discrepancies (5,6). Yet, our study showed that more than half of the lingual practitioners are hesitant to treat complex cases with lingual braces.

Factors that influence orthodontists’ decision to practice or not practice lingual orthodontics, as well as challenges that clinicians currently face with lingual treatment were determined by frequency of response, which are illustrated in Figures 3a, 3b and 4a.

Factors that seemed to have the most influence on a clinician’s decision to practice lingual braces were the following: improved esthetics, practice differentiation and increased case acceptance (Fig. 3a). This finding suggests that patients who would have otherwise declined orthodontic treatment accepted treatment with lingual braces due to its esthetic advantage. Our finding was consistent with other studies which demonstrated that many adult patients would refuse orthodontic treatment with labial appliances and were willing to accept a higher fee for appliances that they deem to be more esthetic (3,14).

Figure 3b illustrates that the challenges most frequently encountered by those who used lingual braces were patient discomfort, cost, longer chair-time and technical difficulties. Technical difficulties listed by the clinicians who offered lingual treatment in their practices were associated with archwire changes, wire bending, difficulties in areas of cingulum, crowding and interbracket distances.

In our study, factors that were identified to have the most influence on practitioner’s decision to not practice lingual orthodontics were the following: technical difficulties, availability of alternative appliances, lack of demand, and patient discomfort (Figure 4a). Despite noTable development of lingual systems in the U.S, technical difficulties and patient discomfort seemed to have remained as commonly recognized disadvantages in lingual orthodontics by both groups. On the contrary, the decision of practitioners to not use lingual braces was largely influenced by lack of demand, whereas only 20% of practitioners who practiced lingual orthodontics reported lack of demand as one of the challenges associated with practicing lingual braces.

Of the orthodontic specialists who did not currently practice lingual braces, many (69%) expressed that they are very likely to incorporate lingual orthodontics in their practices in the future (Fig. 4b). Reinforcing advantages and eliminating challenges associated with lingual orthodontics as identified in this study may present opportunity for more orthodontists who wish to adopt this treatment modality. One of the limitation of our study is the low response rate. The survey questionnaire was sent to 2,200 active U.S. members of the American Association of Orthodontists (AAO). The AAO active members have been randomly divided into 3 groups of 2,200 members in each group. Such surveys are typically sent to only one group of 2,200 members by the AAO to avoid “survey fatigue”. A plausible explanation to the low survey response rate can be due to decreased interest in the orthodontists to participate in the survey. Orthodontists who are busy in their practice may have limited time to respond to such surveys. Survey fatigue can also a reason. It can be speculated that the results of our study could be the same or have varied results if the response rate was higher. The low response rate to such a survey study is beyond the control of the investigators or the survey distributor (AAO). Future studies with a higher response rate need to be done in this area of research to better understand the factors influencing Orthodontists’ preference of adopting lingual orthodontics.

## Conclusions

• Within the limitations of this study, it can be concluded that orthodontists who practiced lingual orthodontics are satisfied, if not very satisfied, with their treatment outcome.

• Some of the factors that influenced clinicians’ decision to practice lingual orthodontics were the following: improved esthetics, practice differentiation and increased case acceptance.

• Some of the factors that influenced practitioner’s decision to not practice lingual orthodontics were the following: technical difficulties, availability of alternative appliances, lack of demand and patient discomfort. Approximately 70% of orthodontists who did not currently practice lingual orthodontics indicated that they are very likely to incorporate lingual braces in their practice in the future.
